# Meiotic Chromosome Stability and Suppression of Crossover Between Non-homologous Chromosomes in x*Brassicoraphanus*, an Intergeneric Allotetraploid Derived From a Cross Between *Brassica rapa* and *Raphanus sativus*

**DOI:** 10.3389/fpls.2020.00851

**Published:** 2020-06-16

**Authors:** Hye Rang Park, Jeong Eun Park, Jung Hyo Kim, Hosub Shin, Seung Hwa Yu, Sehyeok Son, Gibum Yi, Soo-Seong Lee, Hyun Hee Kim, Jin Hoe Huh

**Affiliations:** ^1^Department of Plant Science, Seoul National University, Seoul, South Korea; ^2^Interdisciplinary Program in Agricultural Genomics, Seoul National University, Seoul, South Korea; ^3^Plant Genomics and Breeding Institute, Seoul National University, Seoul, South Korea; ^4^BioBreeding Institute, Ansung, South Korea; ^5^Department of Life Science, Chromosome Research Institute, Sahmyook University, Seoul, South Korea; ^6^Research Institute of Agriculture and Life Sciences, Seoul National University, Seoul, South Korea

**Keywords:** hybrids, polyploidy, meiosis, synapsis, intergeneric hybridization

## Abstract

Hybridization and polyploidization are major driving forces in plant evolution. Allopolyploids can be occasionally formed from a cross between distantly related species but often suffer from chromosome instability and infertility. x*Brassicoraphanus* is an intergeneric allotetraploid (AARR; 2n = 38) derived from a cross between *Brassica rapa* (AA; 2n = 20) and *Raphanus sativus* (RR; 2n = 18). x*Brassicoraphanus* is fertile and genetically stable, while retaining complete sets of both *B. rapa* and *R. sativus* chromosomes. Precise control of meiotic recombination is essential for the production of balanced gametes, and crossovers (COs) must occur exclusively between homologous chromosomes. Many interspecific hybrids have problems with meiotic division at early generations, in which interactions between non-homologous chromosomes often bring about aneuploidy and unbalanced gamete formation. We analyzed meiotic chromosome behaviors in pollen mother cells (PMCs) of allotetraploid and allodiploid F1 individuals of newly synthesized x*Brassicoraphanus*. Allotetraploid x*Brassicoraphanus* PMCs showed a normal diploid-like meiotic behavior. By contrast, allodiploid x*Brassicoraphanus* PMCs displayed abnormal segregation of chromosomes mainly due to the absence of homologous pairs. Notably, during early stages of meiosis I many of allodiploid x*Brassicoraphanus* chromosomes behave independently with few interactions between *B. rapa* and *R. sativus* chromosomes, forming many univalent chromosomes before segregation. Chromosomes were randomly assorted at later stages of meiosis, and tetrads with unequal numbers of chromosomes were formed at completion of meiosis. Immunolocalization of HEI10 protein mediating meiotic recombination revealed that COs were more frequent in synthetic allotetraploid x*Brassicoraphanus* than in allodiploid, but less than in the stabilized line. These findings suggest that structural dissimilarity between *B. rapa* and *R. sativus* chromosomes prevents non-homologous interactions between the parental chromosomes in allotetraploid x*Brassicoraphanus*, allowing normal diploid-like meiosis when homologous pairing partners are present. This study also suggests that CO suppression between non-homologous chromosomes is required for correct meiotic progression in newly synthesized allopolyploids, which is important for the formation of viable gametes and reproductive success in the hybrid progeny.

## Introduction

Hybridization and polyploidization are major driving forces in plant evolution (Van de Peer et al., 2017). It is estimated that most of the extant plant species are polyploids that have undergone whole-genome duplication (WGD) in their evolutionary path (Leebens-Mack et al., 2019). Polyploids can be divided into two classes: ones that undergo the multiplication of a whole set of chromosomes within species (autopolyploids) and the others resulting from hybridization between different species followed by chromosome doubling (allopolyploids). Cytogenetically, autopolyploids have random association among four homologous chromosomes (in tetraploids) leading to tetrasomic segregation during meiosis, whereas allopolyploids have two non-pairing sets of homoeologous chromosomes carrying out disomic segregation (Doyle and Egan, 2010). Allopolyploids can be occasionally formed from a cross between genetically divergent species, for instance, between the individuals that belong to different species or even to different genera. Interspecific hybridization and allopolyploidization are likely to contribute to the emergence of many important crop plants such as oilseed rape (*Brassica napus*), cotton (*Gossypium hirsutum*), tobacco (*Nicotiana tabacum*), wheat (*Triticum aestivum*), sugarcane (*Saccharum officinarum*), and coffee (*Coffea arabica*) (Renny-Byfield and Wendel, 2014). However, many studies report that most synthetic allopolyploids exhibited genetic instability and sterility, the latter of which is mainly caused by meiotic abnormalities during sexual gamete formation (Madlung et al., 2005; Mestiri et al., 2010; Szadkowski et al., 2010, 2011).

Meiosis is the process by which the number of chromosomes in a diploid cell is reduced by half producing haploid gametes that are capable of sexual reproduction. Meiotic cell division consists of two consecutive stages meiosis I and II. In particular, the initial process of meiosis I is important for chromosome assortment and recombination of genetic information. According to a current model, meiotic recombination is initiated by a DNA double-strand break (DSB) and 5′–3′ resection followed by strand invasion to form a displacement loop (D-loop) structure (Hunter, 2015; Mercier et al., 2015; Lambing et al., 2017). Once the D-loop is extended, the second end of DSB can anneal to the displaced strand of the D-loop in a process called second end capture annealing, forming a double Holliday junction (dHJ). Resolution of dHJ can lead to reciprocal recombination through interhomolog strand exchanges known as class I COs. Alternatively, class II COs may occur in a dHJ-independent manner presumably by resolution of D-loops (Hunter, 2015; Mercier et al., 2015). The DSBs that do not produce COs are likely to form noncrossovers (NCOs). NCOs can result either from dissolution of dHJ or from D-loops via synthesis-dependent strand annealing, usually concurrent with gene conversion (Hunter, 2015; Mercier et al., 2015). The process of meiosis is further characterized by synapsis formation, the assembly of synaptonemal complex (SC) and chiasma formation, bringing about genetic diversity during gametogenesis. Particularly, formation of synapsis and crossing-overs between homologous chromosomes are essential for subsequent homologous chromosome co-orientation during meiosis I, producing four haploid gametic cells during meiosis II (Mercier et al., 2015; Lambing et al., 2017). Nondisjunction or failure in bivalent formation impairs reductional segregation, frequently causing aneuploidy in gametes. Lack of chromosome pairing in meiosis of interspecific hybrids is one of the main causes of sterility observed in many synthetic hybrids, which is manifest as a post-zygotic barrier in artificial interspecific hybridization (Dion-Côté and Barbash, 2017). Crossing-over between homologous chromosomes is essential for their co-orientation resulting in proper meiotic chromosome segregation (Stewart and Dawson, 2004).

In early generations of synthetic hybrids non-homologous chromosome pairing, multivalent formation, and chromosome rearrangement are frequently observed, and exert a detrimental effect on the survival of allopolyploid plants (Bomblies et al., 2016; Wendel et al., 2018). Thus, meiosis is critical to the success of sexual reproduction ensuring correct segregation of chromosomes into balanced gametes. During homologous chromosomes are synapsed, the SC is formed at the interface between the chromosomes along the axis. ASYNAPTIC1 (ASY1) and ZIPPER1 (ZYP1) are the lateral and axial elements of meiotic chromosomes, respectively. ASY1 is associated with meiotic chromosomes at early prophase I, and ZYP1 is then deposited at the interface between homologous chromosomes upon synapsis formation (Higgins et al., 2005). HUMAN ENHANCER OF INVASION 10 (HEI10) is a component of ZMM complex (ZIP4, MSH4/5, MER3, MLH1/3) that mediates a meiotic crossover (Chelysheva et al., 2012; Mercier et al., 2015; Gonzalo et al., 2019). The coordinated action of these proteins is crucial for the establishment and progression of synapsis formation and recombination, and abnormal meiosis often results from the lack of proper configuration of these chromatin components.

The Brassicaceae family contains a number of vegetable crops such as broccoli, cabbage, cauliflower, oilseed rape, turnip and radish. Several *Brassica* species are famous for interspecific hybridization to produce allotetraploid plants. For instance, three diploid species *B. rapa* (AA), *B. nigra* (BB), and *B. oleracea* (CC) can be crossed to each other producing allotetraploid species *B. napus* (AACC), *B. juncea* (AABB) and *B. carinata* (BBCC). Such interspecific cross combinations are epitomized by the model of “U’s Triangle,” which first proposed the process by which ancestral diploid *Brassica* species are combined to create novel tetraploid species (Nagaharu and Nagaharu, 1935). In the Brassicaceae family, hybridization between different species can be expanded to the intergeneric level. Since 1826 when intergeneric hybridization between *Brassica* and *Raphanus* was first reported (Prakash et al., 2009), the allotetraploid plants have been sporadically generated but failed to survive due to genetic instability and sterility (Karpechenko, 1924; McNaughton, 1979; Dolstra, 1982). The recently developed x*Brassicoraphanus* (AARR; 2n = 4x = 38) is also synthesized from a cross between *B. rapa* (AA; 2n = 2x = 20) and *Raphanus sativus* (RR; 2n = 2x = 18). Unlike other synthetic allopolyploid plants, x*Brassicoraphanus* displays great fertility and genetic uniformity over successive generations (Lee et al., 2011, 2017).

We assumed that exceptional genetic integrity of x*Brassicoraphanus* should require a reliable and precise control of meiosis, which is critical not only to the production of functional gametes but also to the maintenance of fertility in successive offspring. For this, non-homologous interactions between the parental chromosomes of *B. rapa* and *R. sativus* must be inhibited during meiosis in x*Brassicoraphanus*, which would otherwise cause detrimental chromosome rearrangements resulting in unbalanced gamete formation. In this study, we investigated meiotic chromosome behaviors in pollen mother cells (PMCs) of newly synthesized allodiploid (AR) and allotetraploid (AARR) x*Brassicoraphanus*, while providing a mechanistic insight into the chromosome compatibility for the reproductive success of hybrids formed between distantly related species.

## Materials and Methods

### Plant Materials Production

Seeds of *B. rapa* cv. Chiifu-401-42, *R. sativus* cv. WK10039, and x*Brassicoraphanus* cv. BB1 were sown on 1× Murashige and Skoog (MS) medium (Duchefa, The Netherlands) supplemented with 2% sucrose and 0.8% plant agar (w/v) in a growth chamber under 16 h of fluorescent light at 20 ± 10 μmol m^–2^ s^–1^, at 24°C for 2 weeks. BB1 was derived from microspore culture of a synthetic hybrid of *B. rapa* and *R. sativus*, and maintained for more than ten generations by self-pollination (Lee et al., 2011). The seedlings were vernalized in the 4°C cold chamber for 4 weeks with 16 h of light and 8 h of dark. The plants were transferred to pots in the greenhouse with the same light condition. Synthetic allodiploid x*Brassicoraphanus* were produced by crossing *B. rapa* cv. Chiifu-401-42 as a female parent with *R. sativus* cv. WK10039 as a pollen donor. Floral buds of *B. rapa* prior to anthesis were emasculated and hand-pollinated with *R. sativus* pollen. Thirty-day-old immature hybrid seeds were cultured on MS medium (Duchefa, Netherlands) supplemented with 2% sucrose (w/v) and 0.8% plant agar (w/v). The seeds were vernalized and transferred to the above-described growth conditions. The newly synthesized allodiploid x*Brassicoraphanus* individuals were subjected to chromosome doubling by applying 0.3% colchicine-soaked cotton on the emerging shoot apical meristem for 2 days.

### Flow Cytometry Analysis

Flow cytometry was used to verify the ploidy level (Pfosser et al., 1995). Leaves of *B. rapa* cv. Chiifu-401-42, *R. sativus* cv. WK10039, their synthetic allodiploid and allotetraploid F1 hybrids, and x*Brassicoraphanus* cv. BB1 were subjected to ploidy analysis. Approximately 20 mg of leaves were finely chopped with a clean razor blade in 1 mL of ice-cold Tris-MgCl_2_ buffer (0.2 M Tris, 4 mM MgCl_2_, 0.5% Triton X-100, pH 7.5) in a glass petri dish on ice (Pfosser et al., 1995). Nuclei were isolated and stained in 50 μg L^–1^ of propidium iodide solution with 50 μg L^–1^ of RNase, filtered through a 40 μm cell strainer, and kept on ice. Flow cytometry was performed on a FACS Canto II flow cytometer (BD Biosciences, United States) system with a medium flow rate according to the manufacturer’s protocol. The data were analyzed with the BD FACSDiva software (BD Biosciences, United States). An FL2 detector was used to measure fluorescence, and forward scatter (FSC) and side scatter (SSC) parameters were used for data analysis according to the manufacturer’s instruction. Fluorescence of *B. rapa* and *R. sativus* was used as a reference to assess the ploidy level of resynthesized hybrids.

### Immunofluorescence of α-Tubulin

For detection of α-tubulin, the method of Wang et al. (2010) was adopted with modifications. Anthers were squashed in SuperFrost Plus^TM^ Adhesion (Thermo Fisher Scientific, United States) slides. The PMCs were incubated with monoclonal anti-α-tubulin IgG (Invitrogen, United States) diluted 1:100 for 2 h at 37°C in a moist chamber. The slides were washed and incubated with a FITC-conjugated anti-mouse IgG (Sigma-Aldrich, United States) diluted 1:50 for 2 h at 37°C in a dark chamber. Subsequently slides were washed again and mounted with a mounting medium with 4′, 6-diamidino-2-phenylindole (DAPI; Vector Laboratories, United States). The prepared slides were imaged using a Leica confocal microscope SP8X controlled by Leica LAS X.

### Genome *in situ* Hybridization (GISH) Analysis

Inflorescence was fixed in the Carnoy’s solution (ethanol : glacial acetic acid, 3:1 v/v) for 24 h and stored in 70% ethanol at −20°C until use. The fixed floral buds with 0.8–1.2 mm in length were rinsed in distilled water and stained with 3% aceto-orcein. Anthers were thoroughly washed with distilled water and treated with the enzyme mixture including 2% Cellulase R-10, 1% Macerozyme R-10 (Duchefa Biochemie, Netherlands), 1% Pectinase, and 0.5% Pectolyase Y23 (Sigma-Aldrich, United States) in 150 mM citrate buffer (pH 4.5) for 60–90 min at 37°C. Treated anthers on the SuperFrost Plus^TM^ Adhesion slides were squashed in 60% acetic acid and air-dried. Genomic DNA was isolated from *B. rapa* and *R. sativus* leaves, fragmented by sonication, separated by agarose-gel electrophoresis, and DNA fragments within the range of 200–500 bp were eluted and purified. The fragmented genomic DNA of *B. rapa* and *R. sativus* was labeled with digoxigenin-11-dUTP and biotin-16-dUTP (Roche, Germany) by nick translation, respectively. For GISH with A- and R- genome probes in x*Brassicoraphanus*, the methods of Kwon and Kim (2009) and Belandres et al. (2015) were adopted with modifications. First, chromosome spreads were incubated with digoxigenin- and biotin-labeled probes along with Fluorescein Avidin DCS (diluted 1:100) (Vector Laboratories, United States) at 37°C for 1 h. After washing three times for 10 min each in 4X SSCT, reactions were performed with rhodamine-conjugated sheep anti-digoxigenin antibody (diluted 1:10) (Roche, Germany) and biotinylated-anti-avidin D antibody (diluted 1:100) (Vector Laboratories, United States) at 37°C for 1 h. In the final reaction, dig-rhodamine and biotin-avidin labeled probes were detected with anti-sheep Texas Red antibody (diluted 1:100) and Fluorescein Avidin DCS (diluted 1:100) (Vector Laboratories, United States), respectively. Chromosomes were counterstained with DAPI in Vectashield reagents (Vector Laboratories, United States). Slides were covered with glass coverslips and examined using Axioskop2 microscope equipped with an Axiocam 506 color CCD camera (Zeiss, Germany).

### Immunolocalization of HEI10

The coding sequence of *BrHEI10* gene was amplified from cDNA of young floral buds of *B. rapa* with oligonucleotides 5′-TTAAGAATTCATGAGGTGCAACGCCTGTTGGAGGG and 5′-TTAACTCGAGGAACAGTTGCGGGCGAGAACG, digested with *Eco* RI and *Xho* I, and then cloned into the pET-28a expression vector at corresponding restriction sites (Novagen, United States). The resulting construct was transformed into *Escherichia coli* Rosetta2 (DE3) strains (Novagen, United States). Transformants were grown at 30°C in 1 L of LB medium in the presence of 50 μg mL^–1^ of kanamycin and 50 μg mL^–1^ of chloramphenicol until OD_600_ reached 0.4. Protein expression was induced with 1 mM of isopropyl b-D-thiogalactopyranoside (IPTG) at 16°C for 16 h. Cells were harvested by centrifugation at 6,500 rpm for 15 min at 4°C, and the pellet was resuspended in 100 mL of ice-cold column buffer (50 mM Tris-HCl, pH 7.4, 100 mM NaCl, 10% glycerol, 0.1 mM dithiothreitol, 0.1 mM PMSF). Cells were lysed by sonication for 5 min in ice (output power, 4; duty cycle, 50%; Branson Sonifer 250, Branson, United States). The lysate was subjected to centrifugation at 9,000 rpm for 25 min at 4°C. Inclusion bodies were collected by centrifugation and dissolved in 4 M urea buffer. Protein concentration was estimated using the Coomassie Brilliant Blue R 250 dye-binding method (Bradford, 1976). The purified BrHEI10 protein was used to produce polyclonal antibodies from rabbits by Youngin Frontier (Korea).

A modified version of the method described by Chelysheva et al. (2013) was used to prepare chromosome spreads, for which the fixed inflorescence was rinsed in distilled water and subsequent procedures were essentially the same. For immunolabeling, anti-HEI10 primary antibody was diluted to 1:250 in PBST-BSA buffer and spread onto slides. The slides were covered with parafilm and incubated at 4°C for 39–48 h in a moist chamber, and then washed with PBST. The secondary antibody solution (Goat anti-rabbit IgG H&L, Alexa Fluor 488) diluted to 1:500 was spread on slides and incubated at 37°C for 1 h in a dark moist chamber. After wash with PBST, slides were mounted with a mounting medium with DAPI (Vector Laboratories, United States) to counterstain chromosomes. Photographs were taken using Axioskop2 microscope equipped with an Axiocam 506 color CCD camera (Zeiss, Germany).

### Synteny Analysis

Genome assembly and gene annotation data obtained from the databases of *B. rapa* (Zhang et al., 2018),^1^ *B. oleracea* (Liu et al., 2014; Cunningham et al., 2019),^2^ and *R. sativus* (Jeong et al., 2016)^3^ were subjected to synteny analysis using Synorth (Cheng et al., 2012) with default parameters. The regions containing at least 20 syntenic orthologs were defined as syntenic blocks.

## Results

### Diploid-Like Meiotic Behavior in Synthetic Allotetraploid x*Brassicoraphanus*

**FIGURE 1:**
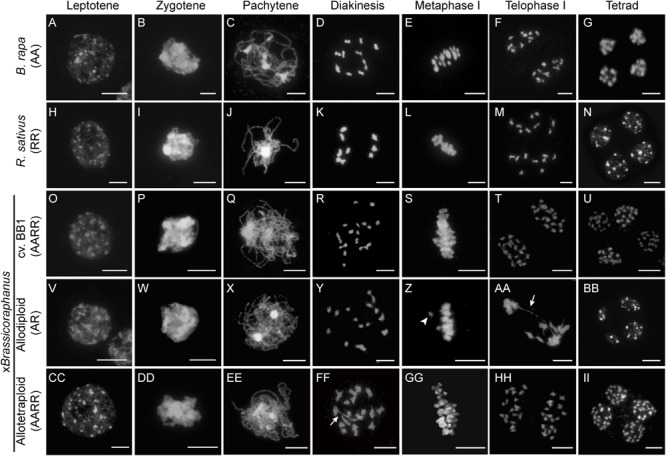
Chromosome behavior during meiosis. The chromosomes in PMCs of *B. rapa* **(A–G)**, *R. sativus* **(H–N)**, BB1 **(O–U)**, and synthetic allodiploid **(V–BB)** and allotetraploid x*Brassicoraphanus* **(CC–II)** were visualized with DAPI staining. Meiotic chromosomes were condensed at leptotene **(A,H,O,V,CC)**, and seen as thin threads at zygotene **(B,I,P,W,DD)**. The synapsis between homologous chromosomes appeared at pachytene **(C,J,Q,EE)**. However, chromosomes were not juxtaposed and only thin chromosome threads were observed at pachytene of synthetic allodiploid x*Brassicoraphanus* **(X)**. Homologous chromosomes were condensed and bivalents were formed at diakinesis **(D,K,R,FF)**. Various numbers of univalents and multivalents were observed in synthetic allodiploid x*Brassicoraphanus* **(Y)**. All chromosomes were aligned on the metaphase plate at metaphase I **(E,L,S,GG)** but unpaired univalent (arrowhead) was sometimes detected in allodiploid x*Brassicoraphanus* **(Z)**. Homologous chromosomes were separated to the opposite poles at telophase I **(F,M,T,AA,HH)**. Chromosome bridges (arrow) were often observed in synthetic allodiploid x*Brassicoraphanus* **(AA)**. Individual chromatids were evenly separated into four microspores at tetrad **(G,N,U,II)**. Unbalanced tetrads were detected in allodiploid x*Brassicoraphanus* **(BB)**. Scale bars = 10 μm.

Many synthetic allopolyploid plants display numeric and structural chromosome aberrations typically caused by abnormal meiosis. Therefore, we first investigated and compared meiotic chromosome behaviors in PMCs of *B. rapa*, *R. sativus*, their synthetic allodiploid and allotetraploid F1 hybrids, and x*Brassicoraphanus* cv. BB1 whose ploidy levels were all confirmed by flow cytometry analysis (Supplementary Figure 1). Normal chromosome behaviors were observed in the entire course of meiosis of *B. rapa*, *R. sativus*, BB1, and synthetic allotetraploid x*Brassicoraphanus* (Figure 1). At leptotene, condensation of meiotic chromosomes was initiated (Figures 1A,H,O,CC). The alignments of homologous chromosomes became prominent at zygotene (Figures 1B,I,P,DD). At pachytene, all chromosomes were closely juxtaposed, preparing for synapsis formation between homologous chromosomes (Figures 1C,J,Q,EE). The chromosomes were condensed into bivalents at diakinesis (Figures 1D,K,R,FF). At metaphase I, bivalents were placed at the metaphase plate (Figures 1E,L,S,GG). The homologous chromosomes were evenly separated at telophase I (Figures 1F,M,T,HH). Finally, four balanced gametes were produced after the second meiotic division in all PMCs (33, 41, 83, and 25 PMCs for *B. rapa*, *R. sativus*, BB1, and allotetraploid x*Brassicoraphanus*, respectively) (Figures 1G,N,U,II). These observations indicate that meiosis occurs normally in synthetic allotetraploid x*Brassicoraphanus* while ensuring faithful chromosome segregation after hybridization between *B. rapa* and *R. sativus*.

Meiotic chromosome behaviors in allodiploid x*Brassicoraphanus* were similar to those of the parents at early stages (Figures 1V,W). However, thinner pachytene chromosomes of all allodiploid x*Brassicoraphanus* PMCs indicate unpaired chromosomes, which would probably lead to rare synapsis formation (59 PMCs; Figure 1X). At diakinesis, bivalents were detected at low frequency and univalents were more frequently observed in allodiploid x*Brassicoraphanus* (Figure 1Y). At metaphase I, two out of 9 PMCs displayed proper alignment of all chromosomes at the metaphase plate, whereas the other seven carried some chromosomes detached from the plate (one isolated chromosome in 3 PMCs, two in 2 PMCs, and three in 2 PMCs) (Figure 1Z). Subsequently, meiotic chromosomes were unequally segregated, and in several occasions (13 out of 20 PMCs), chromosome bridges appeared at anaphase I/telophase I (Figure 1AA). In tetrad, unbalanced gametes with unequal numbers of chromosomes in each microspore were formed at the end of meiosis (Figure 1BB). These observations suggest that meiosis in allodiploid x*Brassicoraphanus* PMCs has a severe defect mostly due to a lack of homologous pairing, albeit some non-homologous interactions still persist as exemplified by bridge formation (Figure 1AA).

### Microtubule Distribution in x*Brassicoraphanus*

**FIGURE 2:**
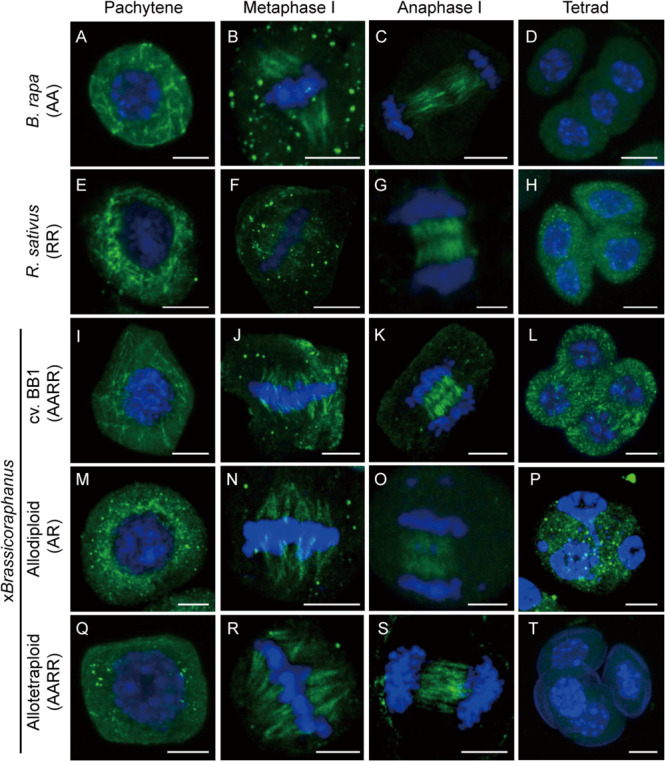
Microtubule distribution during meiosis. Microtubule and chromosome behaviors were observed in PMCs of *B. rapa* **(A–D)**, *R. sativus* **(E–H)**, BB1 **(I–L)**, and synthetic allodiploid **(M–P)** and allotetraploid x*Brassicoraphanus* **(Q–T)**. Microtubules appeared throughout the cytoplasm of the PMC at pachytene **(A,E,I,M,Q)**. Connections between chromosomes and microtubules were displayed at metaphase I **(B,F,J,N,R)**. Phragmoplast microtubules were located between two daughter nuclei in anaphase I **(C,G,K,O,S)**, and four separated daughter nuclei were observed at tetrad **(D,H,L,P,T)**. Microtubules and chromosomes were in green and blue, respectively. Scale bars = 5 μm.

Microtubules are important for the formation of meiotic spindles to support correct segregations of chromosomes. Microtubule dynamics was investigated in *B. rapa*, *R. sativus*, and allodiploid and allotetraploid x*Brassicoraphanus* through immunostaining of α-tubulin at different stages of meiosis (Figure 2). At pachytene, microtubules were organized at the perinuclear zone (Figures 2A,E,I,M,Q). At metaphase I, microtubules were arranged into the spindle structure and attached to kinetochores, engaging a typical bipolar fusiform configuration at the metaphase plate (Figures 2B,F,J,N,R). At anaphase I, microtubules pushed chromosomes toward the opposite poles (Figures 2C,G,K,O,S). Notably, some chromosomes of synthetic allodiploid x*Brassicoraphanus* were not attached to meiotic spindles (Figure 2O). Then interzonal microtubules appeared at the equator forming the phragmoplast. At completion of meiosis, microtubules dissolved and dispersed in the cytoplasm in tetrads (Figures 2D,H,L,P,T). These observations indicate that microtubules behave normally in x*Brassicoraphanus* PMCs.

### Non-homologous Chromosome Associations at Meiosis of Synthetic Allotetraploid x*Brassicoraphanus*

**FIGURE 3:**
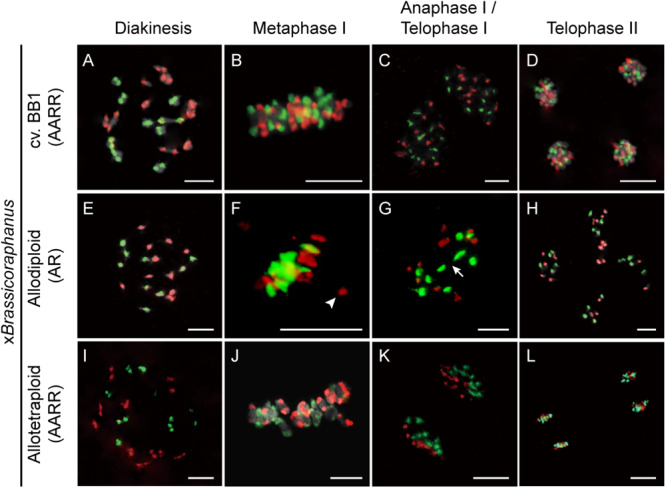
Chromosome identification of x*Brassicoraphanus* by GISH analysis. Distribution of A and R chromosomes were observed in PMCs of BB1 **(A–D)**, and synthetic allodiploid **(E–H)** and allotetraploid x*Brassicoraphanus* **(I–L)**. Nineteen complete bivalents comprising ten from *B. rapa* and nine from *R. sativus* were shown in BB1 and allotetraploid x*Brassicoraphanus*, and homoeologous interactions between A and R chromosomes were scantily detected in allodiploid x*Brassicoraphanus*. The A and R chromosomes are stained in red and green, respectively. Arrow indicates the position of chromosome bridge formed between R chromosomes (Supplementary Figure 3). Arrowhead indicates an isolated chromosome. Scale bars = 10 μm.

Non-homologous chromosome pairing often induces meiotic chromosome aberrations in many resynthesized allopolyploids (Madlung et al., 2005; Mestiri et al., 2010; Szadkowski et al., 2010, 2011). To investigate non-homologous interactions between A and R chromosomes in x*Brassicoraphanus*, GISH analysis was performed during meiosis (Figure 3). Twenty chromosomes of *B. rapa* and 18 chromosomes of *R. sativus* existed in BB1 and synthetic allotetraploid x*Brassicoraphanus* at diakinesis (Figures 3A,I). At diakinesis and metaphase I, 19 bivalents were present in an autosyndetic (A-A or R-R) form, probably with ten A-A bivalents and nine R-R bivalents in synthetic allotetraploid x*Brassicoraphanus* (Figures 3A,B,I,J). At telophase I, chromosomes were correctly segregated, and ten A chromosomes and nine R chromosomes were evenly distributed at each pole in synthetic allotetraploid x*Brassicoraphanus* (Figures 3C,K). At telophase II, chromosomes were evenly segregated to tetrads with ten A and nine R haploid chromosomes, respectively (Figures 3D,L). A-R chromosome associations were unnoticeable during the entire course of meiosis in synthetic allotetraploid x*Brassicoraphanus*. These observations suggest the absence of non-homologous interactions between A and R chromosomes, or very few, if any, which would prevent chromosome rearrangement and aneuploidy in newly synthesized allotetraploid x*Brassicoraphanus.*

In PMCs of allodiploid x*Brassicoraphanus*, ten chromosomes of *B. rapa* and nine chromosomes of *R. sativus* were present, but they mis-segregated at later stages of meiosis (Figures 3E–H). At diakinesis, 0.36 A-A and 0.56 R-R autosyndetic bivalents on average were observed, whereas 1.16 A-R allosyndetic bivalents were present (Table 1 and Supplementary Figure 2). Also, 4.63 A and 3.80 R univalents on average were observed at diakinesis in allodiploid x*Brassicoraphanus* (Table 1). A smaller number of multivalents (0.81 trivalent and 0.78 quadrivalent or more on average) were observed with very few autosyndetics (Table 1). These observations indicate that non-homologous interactions still persist in allodiploid x*Brassicoraphanus*, albeit only 5.1% of PMCs (*n* = 78) contained 19 univalents without chromosome pairing (Figure 3E). At metaphase I, most univalent chromosomes were placed at the metaphase plate but a few were detached as isolated units (Figure 3F). At anaphase I, chromosome bridges were often observed in allodiploid x*Brassicoraphanus* PMCs (Figure 3G and Supplementary Figure 3). At telophase II, A and R chromosomes were randomly segregated to each microspore (Figure 3H). A low frequency of A and R associations suggests that a considerably low level of meiotic recombination likely occur in allodiploid x*Brassicoraphanus*. This also suggests that non-homologous interactions between A and R chromosomes are not preferred during synapsis formation at early stages of meiosis.

**TABLE 1:** Chromosome associations in PMCs of allodiploids at diakinesis as revealed by GISH.

Lines	Total PMCs	I			II				III	≥IV
		I^A^	I^R^	Total	II^AA^	II^RR^	II^AR^	Total		
#20	38	5.18 (0–9)	3.96 (2–7)	9.14 (2–15)	0.39 (0–2)	0.57 (0–1)	1.25 (0–4)	2.21 (0–5)	0.71 (0–2)	0.61 (0–2)
#30	16	4.05 (2–7)	3.15 (0–5)	7.20 (2–12)	0.40 (0–2)	0.60 (0–2)	1.50 (0–5)	2.50 (0–7)	0.95 (0–3)	0.85 (0–2)
#43	24	4.67 (0–10)	4.28 (1–9)	8.94 (2–19)	0.28 (0–2)	0.50 (0–2)	0.72 (0–2)	1.50 (0–4)	0.78 (0–3)	0.89 (0–2)
Average		4.63 ± 0.57	3.80 ± 0.58	8.43 ± 1.07	0.36 ± 0.07	0.56 ± 0.05	1.16 ± 0.4	2.07 ± 0.52	0.81 ± 0.12	0.78 ± 0.15

*I, univalent; II, bivalent; III, trivalent; ≥ IV, more than quardrivalent. I^*A*^ and I^*R*^ indicate univalent belonging to the A and R genomes, respectively. II^*AA*^ and II^*RR*^ indicate autosyndetic bivalents and II^*AR*^ indicates allosyndetic bivalents formed between A and R chromosomes.*

### Suppression of Crossovers in Synthetic Allodiploid x*Brassicoraphanus*

**FIGURE 4:**
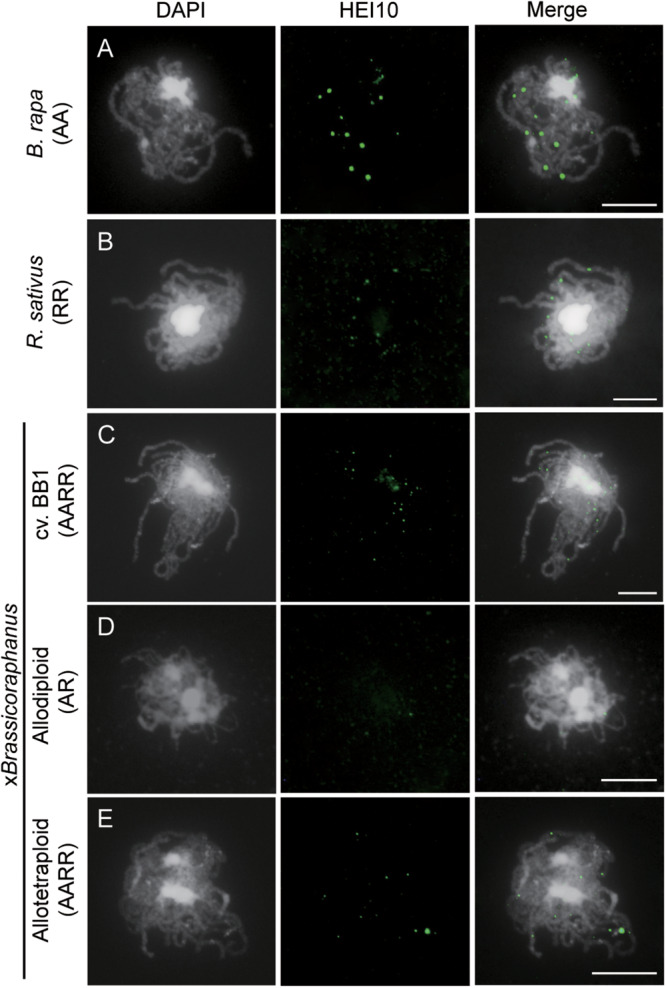
Immunolocalization of HEI10 at pachytene. HEI10 foci were observed in PMCs of *B. rapa* **(A)**, *R. sativus* **(B)**, BB1 **(C)**, synthetic allodiploids **(D)**, and allotetraploid x*Brassicoraphanus* **(E)**. Few HEI10 foci were detected in allodiploid x*Brassicoraphanus*. Chromosomes were labeled with DAPI (white) and HEI10 antibodies (green). The overlay of two signals is shown (Merge). Scale bars = 10 μm.

**FIGURE 5:**
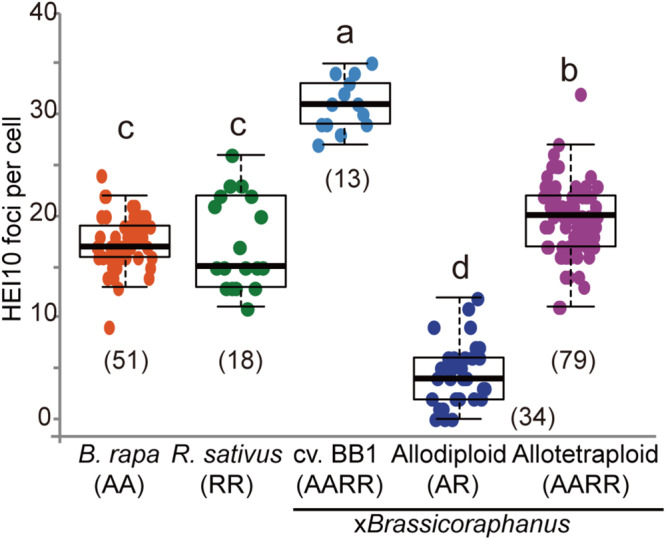
The number of HEI10 foci per PMC at pachytene. The parental species showed similar number of HEI10 foci (17.54 ± 3.48 in *B. rapa* and 17.33 ± 4.51 in *R. sativus*). BB1 represented twice number of HEI10 foci than parental species (30.92 ± 2.53 in BB1) and slightly low number of foci were observed in resynthesized allotetraploid x*Brassicoraphanus* (19.74 ± 3.52). In synthetic allodiploid x*Brassicoraphanus*, only few foci were detected (4.38 ± 2.97). The numbers of observed PMCs were represented in parenthesis. One way analysis of variance (ANOVA) showed differences among treatments (*p* < 2.5e^– 18^) and letters indicate the differences followed by Duncan test (*p* < 0.05).

Formation of COs was investigated by immunolocalization of HEI10 at pachytene of *B. rapa*, *R. sativus*, and synthetic allodiploid and allotetraploid x*Brassicoraphanus*. It is known that HEI10 is essential for transition of early recombination intermediates into final class I COs, which represent the actual sites where strand exchanges and recombination take place (Chelysheva et al., 2012; Gonzalo et al., 2019). To examine intensity and frequency of COs, HEI10 foci were examined in *B. rapa*, *R. sativus*, and synthetic allodiploid and allotetraploid x*Brassicoraphanus* (Figure 4). The average number of HEI10 foci at pachytene was 17.54 in *B. rapa* (*n* = 51 PMCs) and 17.33 in *R. sativus* (*n* = 18 PMCs) (Figure 5). In BB1, 30.92 foci on average were observed (*n* = 13 PMCs), suggesting that an increase in number of COs was attributed to the doubled chromosome number by allopolyploidization. In synthetic allotetraploid x*Brassicoraphanus*, 19.74 HEI10 foci were observed on average (*n* = 79 PMCs) (Figure 5). Interestingly, only 4.38 HEI10 foci on average were detected in allodiploid x*Brassicoraphanus* (*n* = 34 PMCs) (Figure 5), and the HEI10 foci were less conspicuous compared to the parental species and allotetraploid x*Brassicoraphanus* (Figure 4). It is reported that the formations of large and bright HEI10 foci occur only in properly synapsed regions (Grandont et al., 2014), and our observations suggest that a faint HEI10 signal is attributed to unstable synapsis between chromosomes in allodiploid x*Brassicoraphanus*. Also, non-homologous recombination is unlikely to occur in x*Brassicoraphanus* owing to few interactions between A and R chromosomes.

### Structural Divergence of A and R Genomes

**FIGURE 6:**
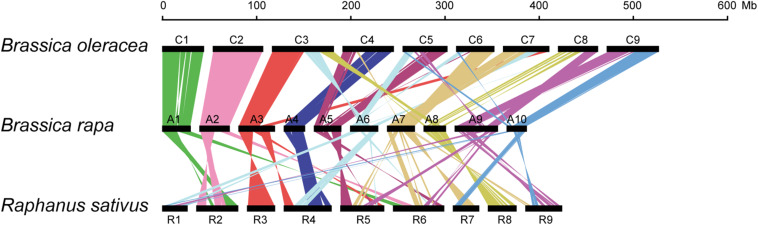
Schematic representation of chromosomal synteny among *B. rapa* (A1–A10), *B. oleracea* (C1–C9) *and R. sativus* (R1–R9). Each pair of syntenic blocks is connected with colored lines.

Formation of allosyndetics in PMCs of allodiploid x*Brassicoraphanus* suggests that chromosomes of *B. rapa* and *R. sativus* share regions similar enough to allow non-homologous interactions. Thus, we conducted synteny analysis to investigate the degree of genome similarity between the species. We identified a total of 339 synteny blocks consisting of 25,054 orthologous gene pairs between A and C genomes of *B. rapa* and *B. oleracea*, and 324 synteny blocks with 17,918 pairs between A and R genomes of *B. rapa* and *R. sativus*. Comparison of synteny blocks revealed that large portions of A1 (76.9%), A2 (71.9%), and A4 (75.9%) chromosomes of *B. rapa* are highly syntenic to those of C1 (76.6%), C2 (73.8%), and C4 (42.7%) of *B. oleracea*, respectively (Figure 6 and Supplementary Table 1). The A genome of *B. rapa* also shares syntenic regions with R genome of *R. sativus* but the similarity is substantially lower. For instance, A3 (29.2%) and A8 (51.7%) are syntenic to R3 (59.9%) and R8 (51.7%), respectively, but the level of similarity is relatively low in other chromosomes (Figure 6 and Supplementary Table 2). Moreover, the R genome appears to be more fragmented from the A genome compared to the C genome of *B. oleracea* (Figure 6). This suggests that the low synteny level, along with structural divergence, is conceivably responsible for the suppression of non-homologous interactions and crossovers between A and R chromosomes in x*Brassicoraphanus*.

## Discussion

Hybridization barriers exist in nature to prevent a gene flow between different species, and can be divided into pre- and post-zygotic stages according to the timing of fertilization. Pre-zygotic barriers prevent fertilization between species, whereas post-zygotic barriers are mechanisms engaged after fertilization that reduce the viability or fertility of hybrid offspring. In particular, hybrid sterility is often associated with a failure in meiosis. Normal meiosis requires the formation of COs between homologous chromosome pairs, and when they are abolished or formed between multiple and/or non-homologous chromosomes, the chromosomes segregate abnormally, resulting in unbalanced gamete formation and reduced fertility (Martinez-Perez and Colaiacovo, 2009).

x*Brassicoraphanus* has a full complement of both parental chromosomes. Unlike many other resynthesized allopolyploids, x*Brassicoraphanus* did not show aneuploidy or apparent chromosome rearrangements, suggesting that COs between non-homologous chromosomes rarely occur during meiosis, despite we cannot completely rule out the possibility that NCOs and gene conversion may occur. Indeed, the number of parental chromosome interactions per PMC in synthetic allodiploid x*Brassicoraphanus* (Table 1) is significantly lower than that of allodiploid *B. napus* (1.16 vs. 3.45) (Cui et al., 2012). In addition, 55.64 and 88.9% of allodiploid chromosomes of x*Brassicoraphanus* and *B. napus* (Szadkowski et al., 2011), respectively, participated in the formation of bivalents or multivalents at early stages of meiotic prophase I (Table 1). We also showed that meiosis in allotetraploid x*Brassicoraphanus* proceeds normally like a diploid cell, albeit A-R chromosome interactions are sporadically observed in allodiploid x*Brassicoraphanus*. This suggests that during meiotic prophase I the chromosome pairing preferentially occurs between the homologous chromosomes of the same progenitor, although non-homologous interactions are also possible when there is no authentic homologous counterpart.

At early stages of meiotic prophase I, homologous chromosomes are aligned in juxtaposition and SCs are formed at the interface between them along the axis, where ASY1, ZYP1 and HEI10 proteins systematically participate in the formation of COs to exchange chromatids. In resynthesized *B. napus*, synapsis is frequently formed between A and C chromosomes (C from *B. oleracea*) via similar segments carried by different chromosomes, and non-homologous recombination results in aneuploidy and interchromosomal rearrangement (Gaeta et al., 2007; Xiong et al., 2011). Such homoeologous regions are still remnant in *B. rapa* and *B. oleracea* genomes although they have diverged several million years ago. For example, A1/C1, A2/C2, and the long arm of A5 and short arm of C4 chromosomes share homology with each other (Parkin et al., 2005). To note, allodiploid x*Brassicoraphanus* formed fewer number of COs (4.38 between A and R; Figure 5) than allodiploid *B. napus* (20.3 between A and C; Grandont et al., 2014). This strongly suggests that interactions between A and R chromosomes are intrinsically inhibited in x*Brassicoraphanus* probably due to a scarcity of homologous regions required for synapsis and recombination.

It is notable that BB1 is fertile producing normal pollen, whereas synthetic allotetraploid x*Brassicoraphanus* F1 is sterile mainly due to aborted pollen formation. This indicates that PMCs of resynthesized allotetraploid x*Brassicoraphanus* are able to perform disomic segregation during meiosis, but the later stage has a developmental defect leading to male sterility. Interestingly, the female gametophyte of resynthesized allotetraploid x*Brassicoraphanus* is functional as it produces viable seeds when pollinated with BB1 as a pollen donor. This suggests that synthetic x*Brassicoraphanus* F1 is mechanistically capable of performing normal meiosis, but its developmental abnormality is manifested only in the male gametophyte. Indeed, the progenitor line of BB1 was initially obtained from a cross between commercial *B. rapa* and *R. sativus* cultivars, and thus has a genetic background different from that of x*Brassicoraphanus* synthesized from *B. rapa* cv. Chiifu-401-42 and *R. sativus* cv. WK10039 whose genome sequences are available. In addition, BB1 was generated by microspore culture in the presence of N-nitroso-N-methylurea (NMU) (Lee et al., 2011), which might have induced mutations of unknown genes that would help escape hybrid incompatibility between different species generally observed in many hybrid individuals (Bomblies and Weigel, 2007). We presume that resynthesized x*Brassicoraphanus* is male-sterile as a default state, possibly caused by incompatibility between the paternal nuclear genome and the maternal cytoplasm, but BB1 has overcome such barriers during the course of artificial hybridization by unknown mechanisms.

Diverse species in the genus *Brassica* are considered to have originated from the same ancestral species after genome triplication, which is approximated to be 9–15 million years ago (Town et al., 2006; Yang et al., 2006; Wang et al., 2011; Cheng et al., 2014). Oilseed rape *B. napus* was formed by hybridization between *B. rapa* and *B. oleracea* approximately 7,500 years ago and supposedly went through abundant homoeologous exchanges (Chalhoub et al., 2014). Recent study also proposed that the genera *Brassica* and *Raphanus* are paraphyletic with a close relationship to each other and predicted that hexaploid progenitor chromosomes were rearranged into nine chromosomes in *R. sativus*, while undergoing differential subgenome fractionation and massive chromosome rearrangement (Jeong et al., 2016). However, according to the genome collinearity, *B. rapa* and *R. sativus* still share numerous syntenic regions across the genome, particularly for chromosomes A3/R3 and A8/R8 (Kitashiba et al., 2014). Despite the presence of syntenic regions between *B. rapa* and *R. sativus* genomes, our observations of crossover suppression in synthetic AR hybrids suggest that rearrangement events have rarely occurred in these chromosomal regions, and thus it is less plausible that they have nearly identical structures or compositions to support non-homologous crossovers. In addition, transposable elements are dispersed throughout the genome, and the frequency and classes greatly vary among species. For instance, it is estimated that *B. rapa* and *R. sativus* have different classes of DNA transposons and retrotransposons differently enriched in their genomes (Mitsui et al., 2015). These transposable elements are expected to have been further diversified and fragmented after speciation, uniquely shaping the genomic landscapes in *B. rapa* and *R. sativus*, even in syntenic regions. Therefore, it is presumed that *B. rapa* and *R. sativus* genomes have gradually lost the similarity in genome structure after speciation and become divergent enough to inhibit A-R chromosome interactions. Such structural differences may allow independent assortment of A and R chromosomes during meiosis, which is conceivably beneficial to the acquisition of meiotic stability in x*Brassicoraphanus*.

Moreover, transposable elements are known to have a strong correlation with meiotic recombination rates in most eukaryotes. In particular, heterochromatic regions that usually contain a high density of transposable elements show strong recombination suppression (Kent et al., 2017). Transposable elements are heavily methylated in general and transcriptionally silenced, and DNA methylation also reinforces genome stability by limiting recombination in higher eukaryotes (Greenberg and Bourc’his, 2019). For example, during meiosis DNA methylation may keep transposable elements-rich regions of the genome from engaging in homology-dependent search and recombination (Zamudio et al., 2015). Therefore, we cannot rule out the possibility that epigenetic factors – particularly DNA methylation – have another profound effect on the inhibition of meiotic recombination between non-homologous but still similar regions of the two progenitor chromosomes.

Interestingly, a newly synthesized F1 allotetraploid of *B. rapa* and *R. sativus* showed a significantly lower recombination rate than genetically stable x*Brassicoraphanus* cv. BB1 (Figure 5). This suggests that immediately after hybridization, meiotic recombination is somewhat suppressed probably due to a conflict in recombination machineries between the two parental genomes. Alternatively, abrupt changes in epigenome landscape and chromatin structure after hybridization may interrupt a proper alignment of homologous chromosomes and crossing-over during meiosis. Investigation of meiotic chromosome behavior and recombination in successive generations will give some important clues to transgenerational progression of genome/epigenome stabilization and its effect on the recombination rate in a newly synthesized hybrid. By increasing the number and repertoire of hybridization combinations and performing an in-depth cytological analysis such as BAC FISH, essential features determining the recombination rate will be more clearly understood at the genome and chromosome levels. In addition, immunolocalization of ZYP1 protein will clearly demonstrate whether synapsis is indeed established and SCs properly assembled between A and R chromosomes in newly synthesized x*Brassicoraphanus*.

Eventually, the in-depth genome study on x*Brassicoraphanus* including genome sequencing and annotation, and transcriptome and epigenome profiling will reveal many fundamental aspects of a hybrid genome resulting from a merger between *B. rapa* and *R. sativus* genomes. This will also facilitate researches on interesting traits unique to the hybrids and its application to the breeding program especially taking advantage of hybrid vigor whose genetic regulatory mechanisms are largely unknown.

## Data Availability Statement

All datasets generated for this study are included in the article/Supplementary Material.

## Author Contributions

HP and JH designed the study. HP, JP, and JK performed the experiments. HP, JP, JK, HS, SY, and JH analyzed the data and wrote the manuscript. HS conducted the synteny analysis. HP, JP, JK, HS, SY, and GY prepared the plant materials. SS helped with the GISH experiment. S-SL provided the plant materials. HK provided the technical assistance in cytological analysis. All authors contributed to the manuscript and approved the submitted version.

## Conflict of Interest

The authors declare that the research was conducted in the absence of any commercial or financial relationships that could be construed as a potential conflict of interest.

## Footnotes

1 http://brassicadb.org

2 http://plants.ensembl.org

3 http://radish-genome.org
