# Supplementary material for: Meiotic Chromosome Stability and Suppression of Crossover Between Non-homologous Chromosomes in xBrassicoraphanus, an Intergeneric Allotetraploid Derived From a Cross Between Brassica rapa and Raphanus sativus
Source: Front Plant Sci. 2020 Jun 16;11:851. doi: 10.3389/fpls.2020.00851 (PMC7309133; doi:10.3389/fpls.2020.00851)
Supplement: Supplementary file 1 [file Data_Sheet_1.PDF]

Supplementary Materials for

Meiotic chromosome stability and suppression of crossover between non-homologous chromosomes in x*Brassicoraphanus*, an intergeneric allotetraploid derived from a cross between *Brassica rapa* and *Raphanus sativus*

Hye Rang Park, Jeong Eun Park, Jung Hyo Kim, Hosub Shin, Seung Hwa Yu, Sehyeok Son, Gibum Yi, Soo-Seong Lee, Hyun Hee Kim and Jin Hoe Huh*

* Corresponding author. E-mail: huhjh@snu.ac.kr

This PDF file includes:

Supplementary Figures 1 to 3

Supplementary Tables 1 and 2

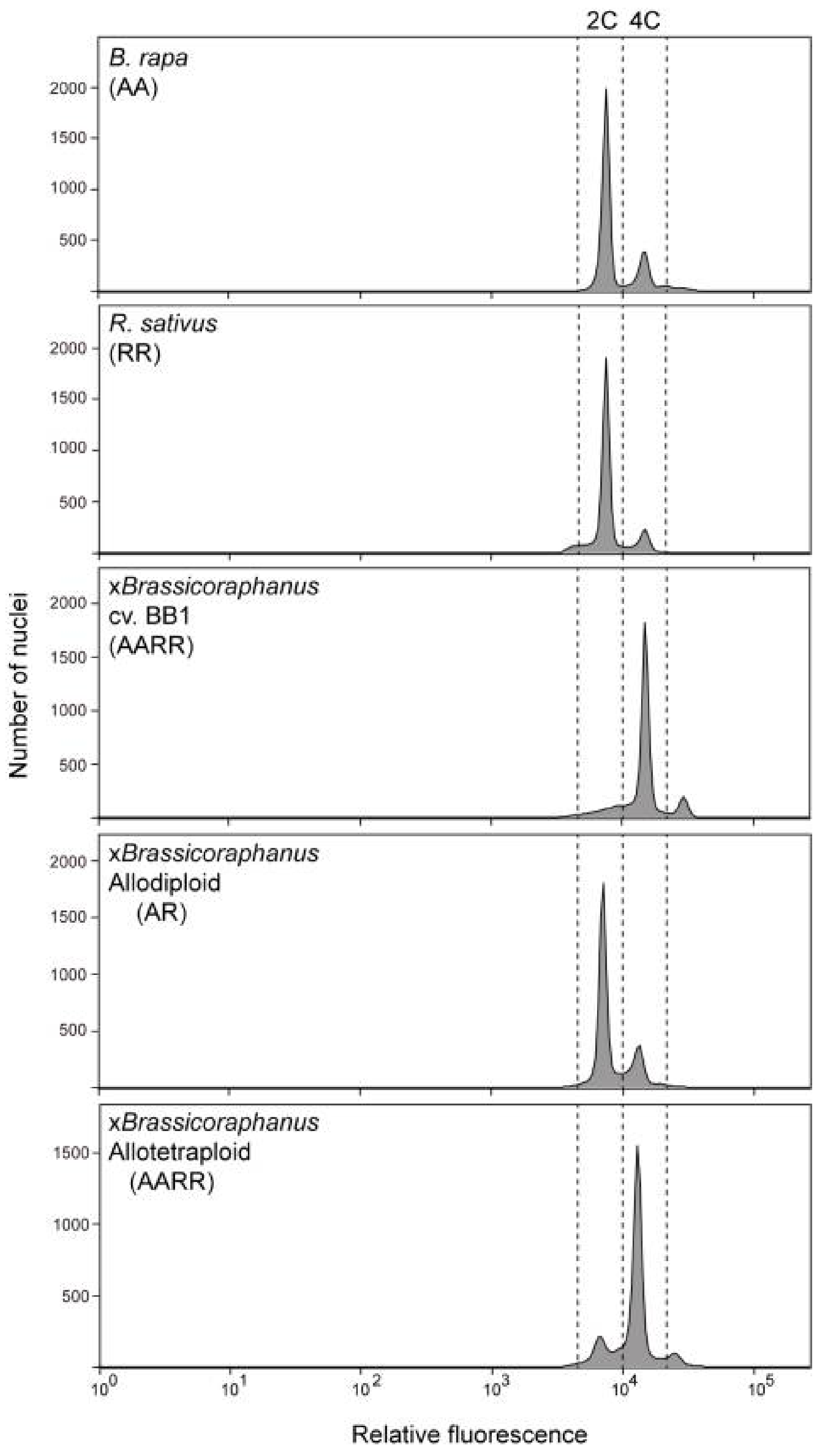

SUPPLEMENTARY FIGURE 1 | Flow cytometric analysis to determine the ploidy level. The x-axis indicates the relative fluorescence and the y-axis represents the number of nuclei. The peaks corresponding to diploids and tetraploids were labeled as 2C and 4C, respectively

This is a provisional file, not the final typeset article

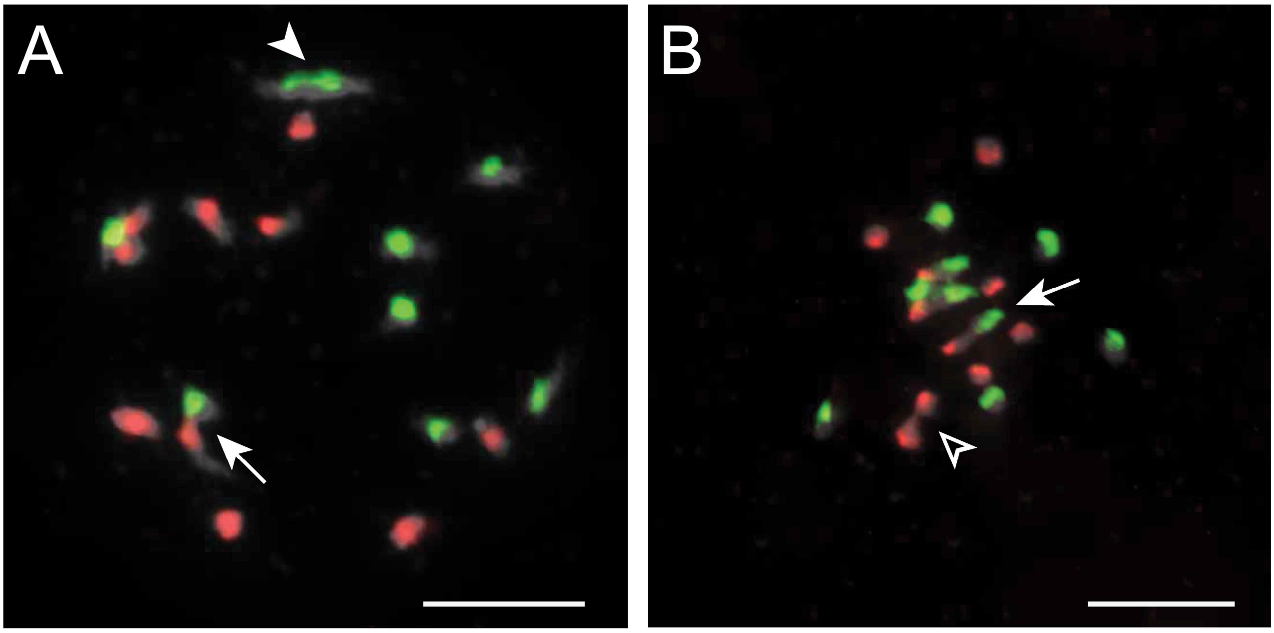

SUPPLEMENTARY FIGURE 2 | Chromosome interactions of synthetic allodiploid x*Brassicoraphanus* at diakinesis. (A) Individual chromosomes of A (red) and R (green) were visualized by GISH. A-R allosyndetic and R-R autosyndetic are denoted with an arrow and closed arrowhead, respectively. (B) A-R allosyndetic and A-A autosyndetic are denoted with an arrow and open arrowhead, respectively. Scale bars = 10 μm.

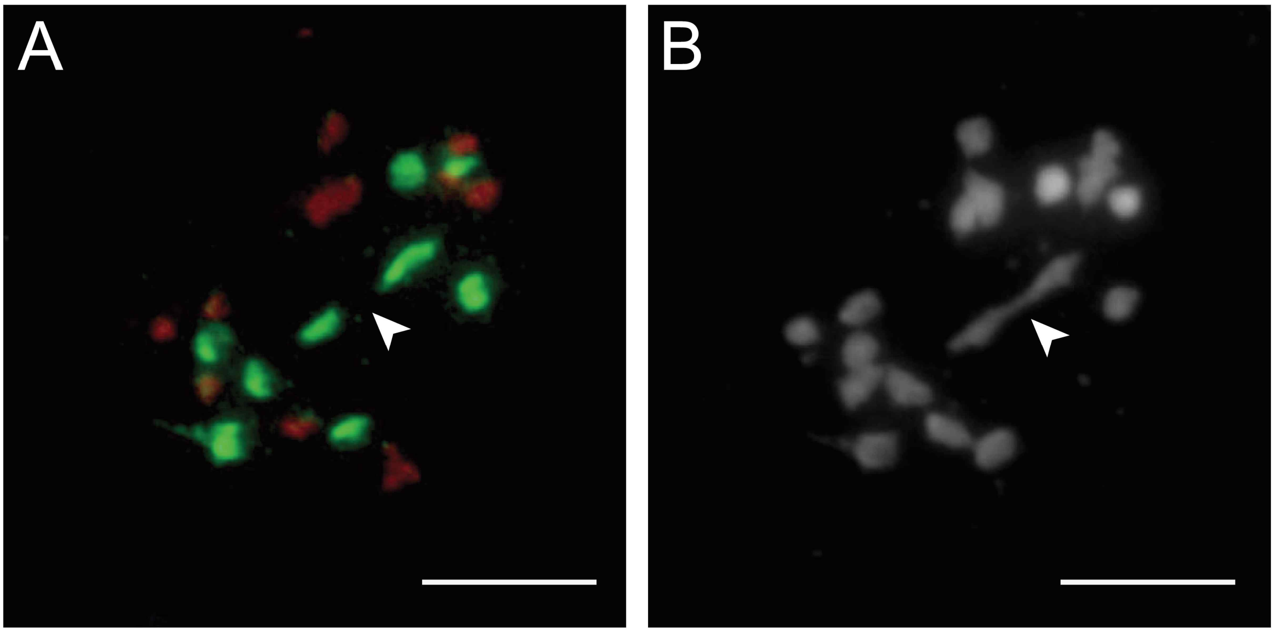

SUPPLEMENTARY FIGURE 3 | Chromosome bridge formation between non-homologous chromosomes at anaphase I in synthetic allodiploid x*Brassicoraphanus*. (A) Individual chromosomes of A (red) and R (green) were visualized by GISH. (B) Chromosomes were DAPI-stained. Arrowhead indicates the position of chromosome bridge. Scale bars = 10 µm.

This is a provisional file, not the final typeset article

Supplementary Table 1 | Lengths of syntenic regions between *B. rapa* and *B. oleracea*

Syntenic region	Length of syntenic region in *B. rapa* chromosomes (bp)	Length of syntenic region in *B. oleracea* chromosomes (bp)	Proportion of syntenic region in *B. rapa* chromosomes (%)	Proportion of syntenic region in *B. oleracea* chromosomes (%)
A01#C1	22,767,858	33,543,135	76.9%	76.6%
A02#C2	22,595,508	39,015,436	71.9%	73.8%
A03#C3	20,217,349	30,293,724	53.0%	46.6%
A03#C7	9,043,567	8,564,227	23.7%	17.7%
A04#C4	16,644,864	22,945,098	75.9%	42.7%
A05#C4	5,878,317	9,221,120	20.6%	17.2%
A05#C5	11,927,554	17,875,187	41.9%	38.1%
A05#C6	1,354,449	2,020,037	4.8%	5.1%
A06#C3	4,561,787	7,582,897	15.6%	11.7%
A06#C5	5,092,254	6,970,648	17.5%	14.9%
A06#C6	973,564	1,873,340	3.3%	4.7%
A06#C7	6,919,063	11,915,166	23.7%	24.6%
A07#C4	848,123	1,651,360	2.9%	3.1%
A07#C6	8,832,450	18,281,773	30.5%	45.9%
A07#C7	7,282,585	12,889,498	25.2%	26.6%
A08#C3	6,324,768	10,399,130	27.5%	16.0%
A08#C8	8,712,277	15,477,427	37.9%	37.1%
A09#C5	4,989,371	7,269,153	11.0%	15.5%
A09#C6	709,721	1,285,576	1.6%	3.2%
A09#C8	10,474,972	12,291,242	23.2%	29.4%
A09#C9	16,189,328	22,576,609	35.9%	41.3%
A10#C5	2,810,223	2,438,055	13.6%	5.2%
A10#C6	484,015	661,620	2.3%	1.7%
A10#C9	10,863,348	18,545,538	52.4%	33.9%

Supplementary Table 2 | Lengths of syntenic regions between *B. rapa* and *R. sativus*

Syntenic region	Length of syntenic region in *B. rapa* chromosomes (bp)	Length of syntenic region in *R. sativus* chromosomes (bp)	Proportion of syntenic region in *B. rapa* chromosomes (%)	Proportion of syntenic region in *R. sativus* chromosomes (%)
A01#R2	11,973,414	11,729,496	40.5%	26.8%
A01#R6	7,909,330	7,339,284	26.7%	13.7%
A02#R2	12,852,126	18,482,314	40.9%	42.2%
A02#R6	5,652,831	5,857,615	18.0%	10.9%
A03#R3	11,144,917	17,450,740	29.2%	59.9%
A03#R4	8,343,072	7,789,236	21.9%	15.6%
A03#R5	5,686,327	5,980,732	14.9%	13.0%
A04#R4	9,919,249	12,060,003	45.2%	24.1%
A05#R5	11,710,515	12,369,028	41.1%	26.9%
A05#R6	6,750,704	6,755,942	23.7%	12.6%
A06#R1	7,414,053	9,078,412	25.4%	34.5%
A06#R4	4,678,609	5,944,910	16.0%	11.9%
A06#R5	3,067,654	2,240,735	10.5%	4.9%
A06#R6	2,948,556	1,785,810	10.1%	3.3%
A07#R5	1,985,591	2,660,121	6.9%	5.8%
A07#R6	4,999,546	6,904,515	17.3%	12.9%
A07#R7	4,342,255	7,492,575	15.0%	27.6%
A07#R9	3,184,513	5,067,138	11.0%	13.2%
A08#R8	11,875,065	15,346,259	51.7%	51.7%
A09#R1	4,997,474	4,826,917	11.1%	18.3%
A09#R5	5,205,950	6,883,107	11.5%	15.0%
A09#R6	3,925,673	5,344,949	8.7%	10.0%
A09#R9	13,296,007	13,182,862	29.4%	34.4%
A10#R1	2,397,444	1,780,759	11.6%	6.8%
A10#R7	5,959,236	7,210,160	28.8%	26.5%
A10#R9	2,807,453	3,882,869	13.5%	10.1%

This is a provisional file, not the final typeset article
